# Individually guided neuromodulation in special operator veterans with symptoms of PTSD and traumatic brain injury: preliminary data from a chart review

**DOI:** 10.3389/fneur.2025.1495034

**Published:** 2025-02-13

**Authors:** B. Christopher Frueh, Celeste Crowder, Alexander Taghva

**Affiliations:** ^1^Department of Psychology, University of Hawaii, Hilo, HI, United States; ^2^Brain Treatment Center, Newport Beach, CA, United States; ^3^Department of Neurosurgery, University of California, San Diego, San Diego, CA, United States; ^4^California Orange County Neurosurgical Associates, Mission Viejo, CA, United States

**Keywords:** neuromodulation, PTSD, MTBI, rTMS, α-rTMS, military, special operations

## Abstract

**Introduction:**

Special operations forces (SOF) are at particular risk of suffering from Posttraumatic Stress Disorder (PTSD) and Traumatic Brain Injury (TBI), and often these two conditions are comorbid, with the inciting event causing both conditions. These conditions present with broad-band electroencephalogram (EEG) abnormalities that may be amenable to neuromodulation.

**Methods:**

This retrospective chart review reports on preliminary safety and clinical response data of individualized neuromodulation in a cohort of SOF veterans suffering from symptoms of PTSD and TBI. 33 male SOF veterans with TBI and PTSD symptoms received α-guided repetitive transcranial magnetic stimulation (α-rTMS) 5 days per week, with the magnetic pulse frequency set to their individual alpha frequency (IAF). Data on clinical scale scores at baseline and conclusion of treatment were extracted, including Rivermead Post-Concussion Questionnaire (RPQ), PTSD Checklist for DSM-5 (PCL-5) and side-effects.

**Results:**

Thirty-three (33) charts containing pre-post scales for at least one of the clinical measures collected were reviewed. TBI symptom severity decreased an average of 54% on the RPQ (*p* < 0.01) and PTSD symptom severity decreased an average of 37.6% on the PCL-5 (*p* < 0.01). For participants with PCL-5 scores above the screening threshold of 33, 69% no longer met clinical criteria for PTSD at the end of the human performance program. Side effects were consistent with those reported for standard TMS, most frequently headache and fatigue.

**Conclusion:**

Significant reductions in TBI clinical symptoms as well as significant decreases in PTSD clinical severity were reported in SOF veterans who underwent α-rTMS. Side effects were equivalent to those observed in normal TMS. Data supports the need for α-rTMS clinical trials in the SOF veteran population to further demonstrate the clinical impact of this approach.

## 1 Introduction

Military service members are at risk for traumatic brain injury as a consequence of exposure to blast, penetrative injuries, and blunt force injuries in the military environments. Over 450,000 military service members have been diagnosed with traumatic brain injury (TBI) in the US military for the time period 2000–2023 (1). Exposure to psychological trauma and physical injuries contribute to the susceptibility of veterans to PTSD (2), as supported by data from 2021 indicating that PTSD affects approximately 25% of veterans who served in Iraq and Afghanistan (3). Due to the extreme nature of their military service, including a higher number of military deployments and specialized training, special operations forces (SOF) may be more acutely impacted by both TBI impairments and PTSD symptoms (4–7). Development of novel treatment options to address TBI and PTSD in SOF veterans is crucially needed. In this preliminary report, data from charts of SOF veterans who underwent personalized neuromodulation in the form of alpha-guided repetitive transcranial magnetic stimulation (α-rTMS) to address this need were reviewed.

Repetitive transcranial magnetic stimulation (rTMS), a form of noninvasive neuromodulation, induces a brief electro-magnetic field, eliciting eddy currents that modulate neuronal oscillations (8). Daily 10.0 Hz stimulation to the left dorsolateral prefrontal cortex has been demonstrated to improve depression symptom severity (9, 10), and rTMS has been cleared by the FDA for use in treatment-resistant depression. A recent systematic review and meta-analysis of rTMS use in mild to moderate TBI populations in 12 randomized controlled trials reported significant improvements in the Rivermead Post-Concussion Symptoms Questionnaire (RPQ), with no significant improvements in cognition and minimal side-effects (11), however it is theorized that circuits disrupted by TBI may be recovered with appropriate TMS application (12).

Functional magnetic resonance imaging (fMRI) and magnetic resonance imaging (MRI) studies of patients with mild TBI have demonstrated reduced default mode network (DMN) connectivity in the posterior cingulate and parietal regions, and increased connectivity in the medial prefrontal cortex [mPFC; (13, 14)]. The DMN is anatomically comprised of mPFC, posterior cingulate cortex (PCC), inferior parietal lobe, lateral temporal cortex and hippocampal formation (15), with research supporting decreased prefrontal DMN connectivity and increased posterior connectivity in PTSD patients (16, 17). These alterations to DMN connectivity also have consequences in cognitive impairment and emotional regulation, issues that trouble veterans long after military service ceases (3).

With specific relevance to this chart review, the EEG rhythm most commonly associated with the DMN is alpha band activity, the dominant cortical rhythm present during eyes-closed resting states, comprised of EEG oscillations occurring between 8 and 13 Hz (18, 19), which has been reported to be altered in PTSD (20). The human alpha rhythms are generally centered around a single peak frequency, known as the individual alpha frequency (IAF), a stable neurophysiological biomarker, with the frequency of the alpha peak positively related to processing speed (21, 22). Alpha activity is linked closely to DMN function (18) and is similarly disrupted and downregulated in TBI and PTSD, respectively (20, 23). Further, reductions in prefrontal alpha band power have been reported to be correlated with increasing PTSD symptom severity (24).

Alpha-guided neuromodulation, known as Magnetic EEG Resonant Therapy (MeRT), or α-rTMS, is an application of rTMS in which treatment frequency is derived from the patient’s resting EEG, with pulse frequency set to the patient’s IAF (25). Stimulation frequency proximity to IAF has been correlated to improved clinical outcomes over 10.0 Hz rTMS therapy in depression, supporting stimulation specificity within the alpha band (26, 27). Additionally, progressive entrainment and synchronization of network activity to stimulation is greater when pulse parameters of rTMS match with IAF (28, 29) reinforcing the support for precision frequency guidance. An open label prospective study using α-rTMS in 16 veterans with severe PTSD reported reductions in PTSD severity scores (30). Additionally, a report published in 2024 in a population of active-duty special operations forces (regularly exposed to TBI), demonstrated a significant decrease in average PTSD scale score (25). The focus of this chart review is to examine the impact of an α-rTMS treatment course on patient symptoms measured by PTSD and TBI clinical scales and side-effects in a veteran SOF population.

## 2 Method

### 2.1 Clinical population

Charts from SOF veterans with self-reported TBI and PTSD symptoms, enrolled in a Human Performance Program at three outpatient Southern California medical clinics, were examined in this retrospective chart review. This program was sponsored by the Special Operations Care Fund (SOCF) and Tomahawk Charitable Solutions non-profit foundations. Intake and personal history forms, clinical scales, therapy notes, treatment notes, and EEG data from those in the program between January 2019 and July 2020 were reviewed. The program consisted of daily α-rTMS treatment for up to 5 days per week and up to 6 weeks (30 sessions in total), with daily side effects data as well as clinical progress notes and scales gathered over the course of the program. α-rTMS safety and therapeutic efficacy as measured by review of clinical scales and notes were the focus of this chart review.

Clinic staff at all three sites were trained to the same protocol for chart review and data extraction. This included identifying the charts of interest, deidentifying all data in charts, extracting data from clinical scales, daily notes, and side effects data, and entering all data into a spreadsheet that was used to populate a database for analyses. Program disqualification included pacemakers, defibrillators, any metal in the head, history of seizures, as well as current benzodiazepine or alcohol use. EEG findings will be discussed in a future publication. This chart review protocol was IRB approved (IRB Study #1286978).

### 2.2 Clinical scales

Participants in the human performance program were administered clinical scales and EEGs at baseline and at final treatment appointments. Assessments included the PTSD Checklist for DSM-5 [PCL-5; (31)], and the Rivermead Post Concussion Symptoms Questionnaire [RPQ; (32)]. The PCL-5 is a 20-item questionnaire that assesses symptoms of PTSD, with each item measured from 0 to 4, and a final sum score ranging from 0 to 80. The screening threshold for the PCL-5 is 33 for identifying probable PTSD (33). The RPQ is a 16-item questionnaire that assesses symptoms of TBI, with each item measured from 0 to 4, and a final sum score ranging from 0 to 64. The PCL-5 and RPQ scales were used for all participants in the program and based on self-report, with high scores indicating severe symptoms.

The Brief Pain Inventory [BPI; (34)] Short Form, and the World Health Organization Disability Assessment Schedule 2.0 (WHODAS 2.0) 36-Item, Self-Administered Version (35) were also collected in the program. The BPI measures pain severity (four items) and interference (seven items), with each item on a scale from 0 to 10, and a final average score computed for each domain. The WHODAS 2.0. measures the burden of disease across 36-items, each scored from 1 to 4, and categorized into six domains. A final complex summary score is calculated to reflect the overall level of disability from 0 to 100. Side effects were reviewed from daily therapy notes, with focus on incidence of headache, fatigue, nausea/dizziness and potential serious adverse events (SAEs), as normally reported in TMS procedures.

### 2.3 α-rTMS human performance program procedure

Participants in the human performance program received α-rTMS 5 days per week, with the magnetic pulse frequency set to their IAF, and pulse amplitude set to 80% of motor threshold. α-rTMS was delivered in 5 s trains, with 45 s intertrain intervals, for a total of 36 trains, or approximately 30 min. α-rTMS treatment frequency calculated from EEGs recorded following every 10 treatment sessions using a Deymed TruScan EEG system and fitted FlexiCAP, with Ag/AgCl electrodes at electrode positions following the international 10–20 system (36). The treatment frequency was measured as the dominant EEG alpha peak frequency in the 8-13 Hz range in a posterior-occipital region of interest (ROI) consisting of P3, P4, Pz, O1, and O2 electrodes, as previously described (25, 30).

Neuromodulation was administered with a Magventure Magpro R30 Stimulator, using a B65 TMS coil positioned over the medial prefrontal cortex (mPFC) corresponding to the FPz EEG location from the standardized international 10–20 electrode placement system. This stimulation site was used in the prior α-rTMS literature (25, 30) and the mPFC has been identified as a promising therapeutic target for TMS in PTSD patients (37). The program consisted of 30 sessions of α-rTMS over 6 weeks, with additional sessions delivered based on clinician judgment. Participants were monitored before and during clinical sessions by licensed clinicians and completed informed consent to receive α-rTMS. All participants underwent treatment with the understanding that they could stop at any time. EEG recordings and clinical evaluations, including clinical scales to assess symptom profiles common in the military population, were obtained prior to the first therapy session, and within 24 h of the final therapy session, as part of normal clinical practice.

### 2.4 Data analysis

Descriptive statistics were reported for demographics, years of service and clinical characteristics. Categorical variables were presented as frequencies and continuous variables as means and standard deviations. Prior to analyses, data was screened for normality of distribution. Paired two-tailed *t*-tests were conducted on baseline and follow-up total scale scores for each measure to investigate the significance of the clinical response to therapy. Significance was set at *p* < 0.05. All analyses were performed using Python 3.8.

## 3 Results

Thirty-six (36) charts with baseline and follow up EEGs were identified for chart review. Charts were included for analysis if they had pre and post RPQ (n = 32) or pre and post PCL-5 (n = 29). Among the 32 with RPQ data, there were 28 who also had PCL-5 data, and one additional participant with PCL-5 data who did not have RPQ, for a total of 33 unique charts analyzed. Lastly, there were 10 charts that did not contain a completed BPI, and 14 that did not contain completed WHODAS scales, hence results from those domains were considered secondary to PCL-5 and RPQ.

All human performance program participants were male, aged 27 to 63 years (mean 43.5, SD = 8.2). Program participants were veterans of the SOF community, with an average service record of 16 years (SD = 8.2, 3–36). Participants received an average of 33 treatment sessions (SD = 10.4). Twelve of the 33 participants reported a previous diagnosis of TBI and PTSD, eight with TBI-only, four with PTSD-only, while six were not formally diagnosed and three did not provide history (n = 9 ‘unknown’). Full analyses stratified by histories of PTSD and/or TBI were not performed due to small sample sizes. Details of demographics and years of military service are provided in Table 1.

**Table 1:** Sample characteristics and scales by time.

Feature	Baseline (*N* = 33)	Follow up (*n* = 33)	Comparison (*t, p*)
Age (*M, SD*)	43.5, 8.2	-	-
Service Years (*M, SD*)	16, 8.2	-	-
Prior TBI and/or PTSD Diagnosis (*n*)	24	-	-
Prior Diagnosis Unknown (*n*)	9	-	-
RPQ (*n* = 32) (*M, SD*)	33.3, 10.3	15.3, 12.3	*t* = −6.6, *p* < 0.001
PCL-5 (*n* = 29) (*M, SD*)	33.8, 18.5	21.1, 16.6	*t* = −3.6, *p* = 0.001
BPI Pain Severity (*n* = 23) (*M, SD*)	3.8, 1.9	2.3, 1.9	*t* = −4.8, *p* < 0.001
BPI Pain Interference (*n* = 23) (*M, SD*)	3.7, 2.3	1.5, 1.8	*t* = −5.6, *p* < 0.001
WHODAS (*n* = 19) (*M, SD*)	27.0, 12.7	16.9, 13.3	*t* = −2.6, *p* = 0.02

**Figure 1:**
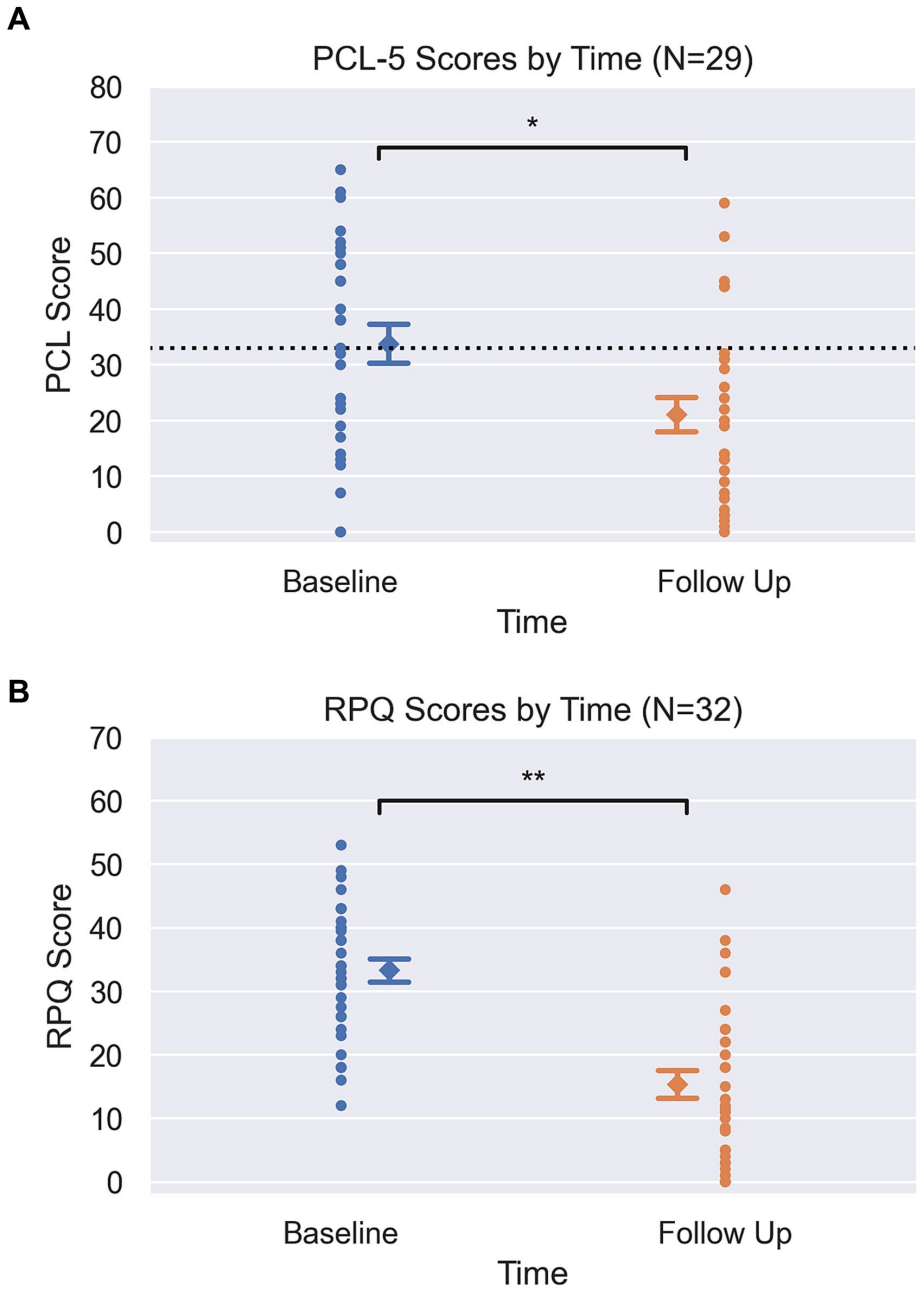
**(A)** Pre and Post PCL scores. Circle markers represent each participant, diamond marker represents mean at each time point with error bars representing +/−1 standard error (PCL pre = 3.4, post = 3.1). Dotted line at y = 33 on PCL graph visualizes clinical screening threshold. Y-axis spans the min and max of each clinical scale (PCL-5: 0–80). *Signifies *p* = 0.001, from pairwise *t*-tests. **(B)** Pre and Post RPQ scores. Circle markers represent each participant, diamond marker represents mean at each time point with error bars representing +/−1 standard error (RPQ pre = 1.8 post = 2.2). Y-axis spans the min and max of each clinical scale (RPQ: 0–64). **Signifies *p* < 0.001, from pairwise *t*-tests.

As clearly demonstrated in Figures 1A,B, RPQ and PCL-5 scores both showed significant changes between baseline and follow up (completion of program). Mean RPQ scores reduced by 54%, from 33.3 to 15.3 (*t* = −6.6, *p* < 0.001, *d* = 1.58) (Table 1). There was a 37.6% reduction in mean PCL-5 score, from 33.8 to 21.1 (*t* = −3.6, *p* = 0.001, *d* = 0.72). Sixteen participants had a baseline PCL-5 score greater than the PTSD screening threshold of 33, with an average score of 47.9 (SD = 9.2, 33–65). At the end of the human performance program, 11 of these 16 participants (69%) had scores below the screening threshold indicating probable PTSD as reflected by a score less than 33, with a group average post-treatment PCL-5 score of 18.5 (SD = 11.7, 0–32). Previously reported diagnosis was unrelated to the change in scores from baseline to follow-up on RPQ (*p* = 0.18) and PCL-5 (*p* = 0.31). In a subset of charts where data was obtained, pain severity and interference sub-scores from the BPI (n = 23) were seen to decrease by 39.5 and 59.5% (respectively), and WHODAS scores (n = 19) decreased by 36% following treatment (Table 1).

The α-rTMS treatment was well-tolerated according to the charts, and side effects were consistent with the level of those reported in similar applications of rTMS (33). Side effects were generally transiently experienced throughout and resolved by the end of the program, specifically at the end of the program 9% (3 of 33) of participants reported headache, 6% (2 of 33) reported fatigue, and 3% (1 of 33) reported nausea/dizziness. Critically, no seizures or other serious adverse events were reported in charts, and no participants discontinued treatment due to side effects.

## 4 Discussion

This retrospective chart review assessed the clinical response and safety of veterans in the Special Operations community suffering from symptoms of TBI and PTSD to α-rTMS treatment delivered as part of a human performance program. Both TBI and PTSD are notably difficult to treat in veterans, and are more prevalent in SOF than in conventional forces, making this population vulnerable to potential long-term impairments across multiple domains of functioning (4). In this chart review we found a significant decline in TBI and PTSD symptom scales, 54 and 37.6%, respectively. Notably, in this chart review 69% of SOF individuals with a PCL-5 score above screening thresholds at baseline no longer met clinical criteria by the end of the program, in contrast to conventional rTMS findings reporting 46.1% of PTSD patients no longer meeting clinical criteria at the end of treatment in non-SOF military veterans with PTSD (33). α-rTMS treatment was well-tolerated, with side effects consistent with those normally reported with the administration of rTMS which highlights the safety profile of α-rTMS. Moreover, significant reductions in pain and improvements in quality of life were also identified, albeit in a smaller sub-sample.

There are limitations to this chart review to be addressed in future studies. The small sample size and lack of homogeneity in subject diagnostic history without verification and without evaluation of consistency of the extracted data increases variance within the population and may contribute to the strength of the differences reported. Small sample size further precluded analysis of the effect of clinical site and additional covariates. Further stratification of histories of PTSD and/or TBI in a larger population may be informative. In addition, there is need for a double-blind sham-controlled studies of α-rTMS in TBI and PTSD populations, respectively. The DMN has been largely implicated in TBI and PTSD symptom pathology, and α-rTMS may be positively impacting network connectivity in this population, however this data was not available in the present chart review. Future studies including resting state MRI to measure pre-post resting activation of DMN structures such as the mPFC and PCC may further elucidate the impact of α-rTMS on DMN function.

Functioning as the “tip of the spear,” with multiple deployments, high operational tempos, and extreme exposure to blast waves in training and combat, special operations forces are acutely affected by TBI impairments and PTSD symptoms (4–6) and are at higher risk of suicide than most other military occupational specialties (38). Noninvasive neuromodulation provides the opportunity to positively entrain neuronal function in populations without the use of long-term pharmacological treatments, potentially addressing patient-specific cortical network disruption through a personalized application of rTMS in contrast to generalized neurotransmitter effects. Additionally, therapeutic outcomes of α-rTMS have been reported to occur within the first few weeks of therapy in both this chart review and previous reports (25, 30), whereas medications have significant side effects and may require months to take effect. Likewise, both pharmacotherapies and psychotherapy have efficacies lower than those reported herein (39, 40). Novel treatments beyond traditional psychiatric medications and psychotherapies for this unique population are urgently needed. Such treatments may treat not only downstream effects of brain injuries at late- or post-career, but may also help mitigate damage at earlier stages of development and thereby improve operational functioning and career longevity in active-duty SOF. Despite the absence of α-rTMS clinical trials for PTSD and TBI in the peer-reviewed literature, preliminary evidence is quite encouraging, and more rigorous randomized clinical trials are underway for the use of α-rTMS in PTSD symptoms and TBI impairments in both active-duty and retired SOF.

## Data Availability

The raw data supporting the conclusions of this article will be made available by the authors, without undue reservation.
