# Correction: Individually guided neuromodulation in special operator veterans with symptoms of PTSD and traumatic brain injury: preliminary data from a chart review

**DOI:** 10.3389/fneur.2025.1683987

**Published:** 2025-10-07

**Authors:** B. Christopher Frueh, Celeste Crowder, Alexander Taghva

**Affiliations:** ^1^Department of Psychology, University of Hawaii, Hilo, HI, United States; ^2^Brain Treatment Center, Newport Beach, CA, United States; ^3^Department of Neurosurgery, University of California, San Diego, San Diego, CA, United States; ^4^California Orange County Neurosurgical Associates, Mission Viejo, CA, United States

**Keywords:** neuromodulation, PTSD, MTBI, rTMS, α-rTMS, military, special operations

In the published article, there was an error in affiliation 2 Instead of “Department of Psychology, University of California, Los Angeles, Los Angeles, CA, United States”, it should be Brain Treatment Center, Newport Beach, CA, United States

There was an error in the **Funding** statement. The **Funding** statement was amended from ‘The author(s) declare that no financial support was received for the research, authorship, and/or publication of this article.' to

‘The authors declare financial support was received for the research and/or publication of this article. The program on which the research was funded by Special Operations Care Fund (SOCF) and Tomahawk Charitable Solutions non-profit foundations. The funders were not involved in the chart review, analysis, interpretation of data, the writing of this article, or the decision to submit it for publication.'

The original article has been updated.

